# Low False-Positives in an mLumin-Based Bimolecular Fluorescence Complementation System with a Bicistronic Expression Vector

**DOI:** 10.3390/s140203284

**Published:** 2014-02-19

**Authors:** Shun Liu, Xiangyong Li, Jie Yang, Zhihong Zhang

**Affiliations:** 1 Britton Chance Center for Biomedical Photonics, Wuhan National Laboratory for Optoelectronics, Huazhong University of Science and Technology, Wuhan 430074, China; E-Mails: liushun@ibp.hust.edu.cn (S.L.); xlxyongli@gmail.com (X.L.); yangjie@mail.hust.edu.cn (J.Y.); 2 Department of Biology, Dezhou University, Dezhou 253023, China

**Keywords:** protein-protein interaction, bimolecular fluorescence complementation (BiFC), expression vector, false positive, fluorescent protein

## Abstract

The simplicity and sensitivity of the bimolecular fluorescence complementation (BiFC) assay make it a powerful tool to investigate protein-protein interactions (PPIs) in living cells. However, non-specific association of the fluorescent protein fragments in a BiFC system can complicate evaluation of PPIs. Here, we introduced a bicistronic expression vector, pBudCE4.1, into an mLumin-based BiFC system, denoted as the BEVL-BiFC system. The BEVL-BiFC system achieved a 25-fold contrast in BiFC efficiency between positive (Fos/Jun) and negative (ΔFos/Jun) PPIs. The high BiFC efficiency was due to a low false-positive rate, where less than 2% of cells displayed BiFC in the negative control. K-Ras and its interactive proteins, Ras binding domain (RBD) of Raf-1 and Grb2 were used to confirm the accuracy of the BEVL-BiFC system. The results also provide direct evidence in individual cells that post-translational modification of K-Ras and its localization at the plasma membrane (PM) were not essential for the interaction of K-Ras and Raf-1, whereas the interaction of Grb2 and K-Ras did depend on the PM localization of K-Ras. Taken together, the BEVL-BiFC system was developed to reduce the false-positive phenomenon in BiFC assays, resulting in more robust and accurate measurement of PPIs in living cells.

## 1. Introduction

Protein-protein interactions (PPIs) play important roles in many cellular processes. To visualise the mechanisms and function roles of PPIs directly, various methods such as bimolecular fluorescence complementation (BiFC) [1,2], and fluorescence resonance energy transfer (FRET) [3], have been developed. Among the two common methods, the BiFC assay is a useful tool to study PPIs in living cells that has been widely used in the past decade [4,5]. Due to the simplicity and sensitivity of the BiFC assay [6], it has been used in the investigation of subcellular localization of PPIs and their regulation mechanisms in living cells, especially the PPIs occurring on the cell membrane or with weak affinity [7–10]. BiFC typically involves using genetic techniques to split a fluorescent protein (e.g., Citrine and Venus [11], and mLumin [12], generally denoted as FP) into two fragments and fusing the respective fragments to two other proteins of interest that will be assessed for interaction. Usually, two plasmids encoding each of the two fusion proteins are then cotransfected into living cells. In theory, if the two proteins of interest interact, the two nonfluorescent fragments of the FP are brought into close proximity and fold into one intact FP [13]. If the proteins of interest do not interact, the fused FP fragments also do not interact and thus do not reconstitute to an intact FP, and no detectable FP signal is detected.

Besides interaction between the proteins of interest, BiFC can occur by spontaneous association of the FP fragments [9,14]. Methods to deal with non-specific BiFC include two different approaches: (1) using a lower concentration of plasmids to reduce the expression of the fusion proteins and decrease the chance of spontaneous association of FP fragments [8]; (2) using mutation technology by replacing some critical amino acid of the split fluoresent protein to reduce self-assembly can increase signal to noise ratios in the Venus-based BiFC system [15,16]. However, rigorous controls should be used to distinguish between true- and false-positive PPI due to the risk of non-specific BiFC [13]. Thus, while non-specific FP fragment associations can be mitigated by ensuring that the proteins of interest are expressed at lower concentrations, the false positive risks of BiFC assay still exist, which may confound identification of unknown or weak PPIs.

In recent years, many modifications and enhancements to BiFC assay have been developed [1,2,17]. Previously, we reported a novel far-red BiFC system based on mLumin, which enables BiFC analysis of PPIs at 37 °C in living cells [12]. Furthermore, the combination of mLumin with Cerulean- and Venus-based BiFC systems achieved simultaneous visualization of three pairs of PPIs in the same cell. mLumin, a bright monomeric far-red FP with an emission maximum of 621 nm, has the potential to extend BiFC assay of PPIs into living small animals [12,18,19]. However, the false-positive phenomenon, brought by spontaneous association of FP fragments, still exists in the mLumin-based BiFC system. pBudCE4.1, a bicistronic expression vector with a CMV promoter and EF-1α promoter, has been widely used to eliminate variable expression of two genes in the same mammalian cells. In this study, we developed an mLumin-based BIFC system with a bicistronic expression vector, denoted as BEVL-BiFC system, which provided a useful tool to decrease the false-positive phenomenon in BiFC assay.

## 2. Experimental Section

### 2.1. Construction of the Bicistronic Expression Vectors

RBD (Ras binding domain of Raf1, 51–131) was amplified using PCR following reverse transcription of RNA extracted from Hela cells. All the PCR primers used in this paper are listed in the Supporting Information. For construction of plasmids pBud-Ln-Fos(ΔFos)-Lc-Jun and pBud-Ln-Fos(ΔFos)-Jun-Lc (Figure S1 in the Supporting Information), intermediate plasmids Ln-Fos and Ln-ΔFos were constructed by generating the fusion of bFos or bΔFos and mLumin (1–151) (Ln) in which bFos or bΔFos were fused to downstream of Ln into the *HindIII* and *BamHI* sites of pBudCE4.1. Fusion of bJun and mLumin (151–233) (Lc) in which bJun was fused either downstream or upstream of Lc was cloned into the *NotI* and *MluI* sites of pBudCE4.1. For construction of plasmids pBud-Ln-RBD-Lc-KRas (K-Ras 12v or K-Ras C185S), the PCR product of RBD was fused downstream of Ln and was inserted using the *HindIII* and *BamHI* sites of pBudCE4.1. K-Ras (K-Ras 12v or K-Ras C185S) was cloned downstream of Lc in *XhoI* and *MluI* sites.

### 2.2. Cells Culture and Transfection

COS-7 cells were maintained in Dulbecco's modified Eagle's medium (DMEM) supplemented with 10% newborn calf serum (NCS), 100 U/mL penicillin, and 100 mg/mL streptomycin in a humidified incubator at 37 °C with 5% CO_2_. The dual protein expression vectors (100 ng) were transfected into COS-7 cells along with the internal control pmCerulean-C1 (30 ng) using Lipofectamine^TM^ 2000 (Invitrogen, Carlsbad, CA, USA)

### 2.3. Fluorescent Microscopy and Image Processing

16 h after cotransfection, fluorescent signals from mLumin and mCerulean in COS-7 cells were analyzed using a wide-field fluorescence microscope or a confocal laser scanning microscopy (for detailed parameters, see Methods in Supporting Information). Image J [20] was used to quantify the fluorescence intensity of cells. BiFC efficiency was calculated using the fluorescence intensity ratio of mLumin to mCerulean in cells expressing internal control mCerulean. Statistical results of BiFC efficiency were from three independent experiments in which more than 100 cells were analyzed. All BiFC efficiency values are given as mean ± S.D. and the statistical significance was evaluated using a two-tailed Student's t-test (*, P<0.001; n.s., no significant).

## 3. Results and Discussion

### 3.1. Evaluation of the BiFC Efficiency and False-Positive Rate of BEVL-BiFC System

**Figure 1.**
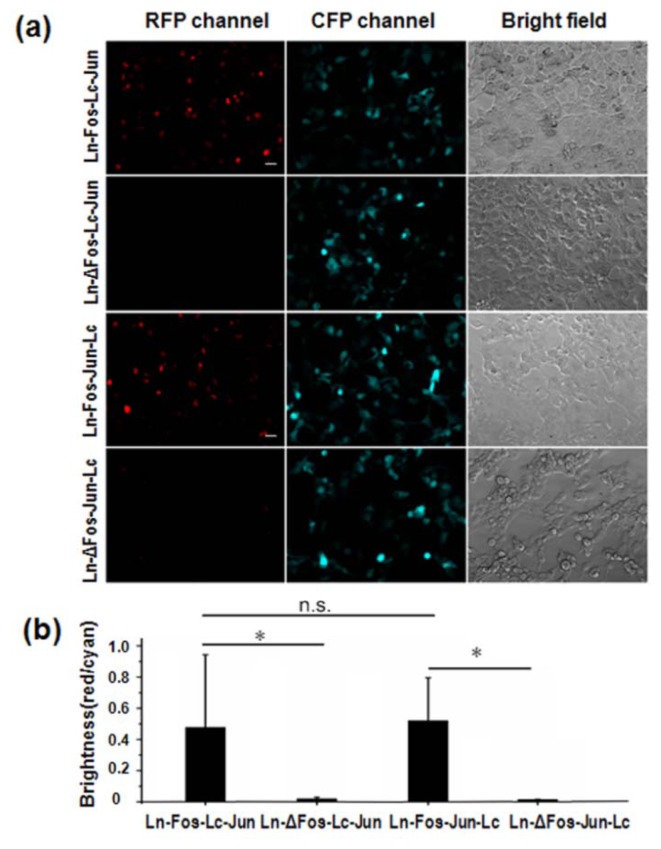
BiFC efficiency of the mLumin-based BiFC system with a bicistronic expression vector. (**a**) Fluorescence imaging and (**b**) BiFC efficiency analysis of COS-7 cells cotransfected with pBud-Ln-Fos(ΔFos)-Lc-Jun or pBud-Ln-Fos(ΔFos)-Jun-Lc and pmCerulean-C1. Scale bar = 20 μm.

Here, the known positive and negative control of PPI, Fos/Jun and ΔFos/Jun [11], were used to evaluate BiFC efficiency and the false-positive rate of the BEVL-BiFC system. Fluorescence imaging data revealed a strong BiFC signal in either pBud-Ln-Fos-Lc-Jun or pBud-Ln-Fos-Jun-Lc transfected cells (Figure 1a). In comparison, there was little BiFC signal in cells transfected with pBud-Ln-ΔFos-Lc-Jun or pBud-Ln-ΔFos-Jun-Lc. The BEVL-BiFC system achieved ∼25-fold contrast in BiFC efficiency between Fos/Jun and ΔFos/Jun (Figure 1b), which is a higher contrast than previously reported BiFC assays [11,12]. The high contrast in BiFC efficiency between the positive and negative control was mainly due to the decreased BiFC signal in the negative control (ΔFos/Jun), where less than 2% cells had detectable BiFC signal. Even as the amount of dual expression vector was increased to 300 ng from 100 ng for cell transfection, the BEVL-BiFC system still maintained a low false-positive rate in the negative control. We also found that BiFC efficiency and the low false-positive rates of the BEVL-BiFC system was not affected whether Jun was on upstream or downstream of C-terminal fragment of mLumin.

### 3.2. Detection of the Interaction between K-Ras and RBD Using BEVL-BiFC System

To verify the robustness of the BEVL-BiFC system in the detection and analysis of PPIs, we examined the plasma membrane (PM) localized protein K-Ras and RBD as a PPI model. K-Ras is usually anchored to cell membranes because of the presence of an isoprenyl group on its C-terminus (Figure S2 in the Supporting Information). As a small GTPase, K-Ras is phosphorylated by guanine nucleotide exchange factors (GEFs) when upstream signal proteins are activated, and then K-Ras transfers the signal to downstream effector proteins (e.g., Raf-1). K-Ras acts as a molecular on/off switch, conversed between active K-Ras bound with GTP (K-Ras-GTP) and inactive K-Ras bound with GDP (K-Ras-GDP). Using immunoblotting and FRET imaging approaches [21], it has been reported that K-Ras bound with GTP is critical for high affinity interaction between K-Ras and RBD. However, these approaches are not efficient to detect transient and weak PPIs in living cells, resulting in confusion whether inactive K-Ras interacts with RBD or not.

**Figure 2.**
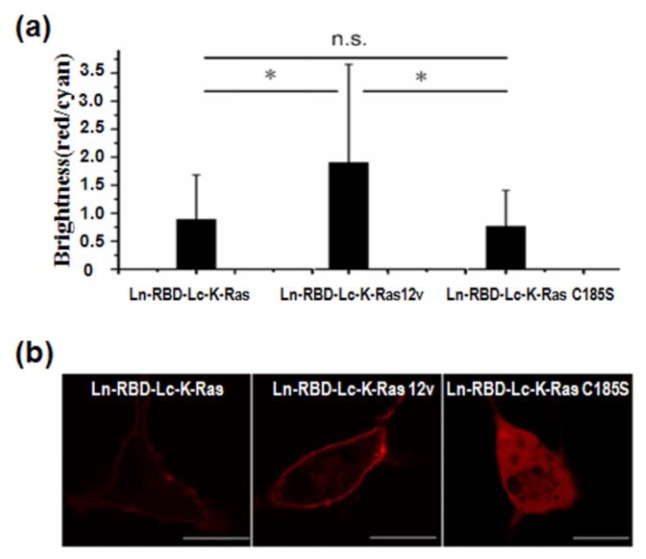
BiFC assays of interaction between K-Ras and RBD. (**a**) BiFC efficiency analysis and (**b**) confocal imaging of cells cotransfected with pBud-Ln-Grb2-Lc-K-Ras (K-Ras 12v or K-Ras C185S) and pmCerulean-C1. Scale bar = 20 μm.

We used the BEVL-BiFC system to investigate whether the interaction between K-Ras and RBD depended on the GTP-bound form of K-Ras and its PM localization. This study was performed with two controls: (1) K-Ras 12v, a constitutively active form of K-Ras that preferentially binds to its effectors; and (2) K-Ras C185S, a mutant with Cys185 replaced with serine to abolish farnesylation of K-Ras. COS-7 cells cotransfected with pBud-Ln-RBD-Lc-K-Ras and pmCerulean-C1 were cultured without serum to keep most of K-Ras in its inactivated form K-Ras-GDP [22]. As shown in Figure 2, BiFC was observed in all groups. RBD and K-Ras 12v displayed the highest BiFC efficiency, which was 2-fold that of RBD/K-Ras and RBD/K-Ras C185S interaction. However, there was no significant difference between the BiFC efficiency of RBD/K-Ras and RBD/K-Ras C185S interaction. Confocal imaging data showed that the interaction between K-Ras (or K-Ras 12v) and RBD mainly occurred at PM (Figure 2b).

Even for non-farnesylated K-Ras C185S, which lost its PM anchor ability, BiFC signal was still detectable in the cytoplasm of cells. These results provided direct evidence in living cells that a certain level of weak interaction between RBD and inactive K-Ras existed in the inactivated cells and this PPI was independent on the farnesylation of K-Ras and its PM localization.

### 3.3. Detection of the Interaction between K-Ras and Grb2 Using the BEVL-BiFC System

**Figure 3.**
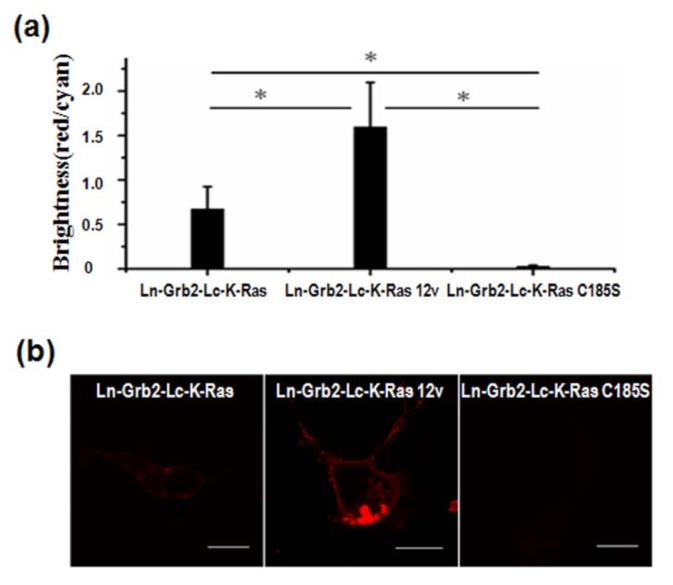
BiFC assays of interaction between K-Ras and Grb2. (**a**) BiFC efficiency analysis and (**b**) confocal imaging of cells cotransfected with pBud-Ln-Grb2-Lc-K-Ras (K-Ras 12v or K-Ras C185S) and pmCerulean-C1. Scale bar = 20 μm.

To confirm the RBD/K-Ras C185S interaction was not due to the false positives induced by the BEVL-BiFC system, we used Grb2 as an experimental control. Like Raf-1, Grb2 also localized in the cytoplasm in non-activated cells and was recruited to the PM by activation signals (Figure S3 in the Supporting Information). Grb2 associated with GEF member (e.g., Sos1) to form a complex that can bind and activate K-Ras on the PM. Thus, PM localization of K-Ras is crucial for Grb2 complex interaction with K-Ras-GDP and its conversion to the GTP-bound form. The dual protein expression vector pBud-Ln-Grb2-Lc-K-Ras (K-Ras 12v or K-Ras C185S) was cotransfected with pmCerulean-C1 into COS-7 cells, using serum-starvation to keep most of the K-Ras in the GDP-bound form. As shown in Figure 3, the strongest BiFC signal was produced by Grb2/K-Ras 12v interaction, a weaker BiFC signal was generated by Grb2/K-Ras interaction, and no BiFC signal was observed in the cells transfected with pBud-Ln-Grb2-Lc-K-Ras C185S. BiFC efficiency ratios of Grb2 interaction with K-Ras, K-Ras 12v and K-Ras C185S were 1:2.4:0.04 (Figure 3a), indicating that PM localization of K-Ras was necessary for interaction between K-Ras and Grb2 complexes. This also indicates that the BiFC signal detected in the cytoplasm of cells transfected with pBud-Ln-RBD-Lc-K-Ras C185S was indeed due to interaction between RBD and K-Ras C185S rather than a false-positive signal.

## 4. Conclusions/Outlook

In this study, a bicistronic expression vector, pBudCE4.1, was introduced into an mLumin-based BiFC system, resulting in reduced false-positive rates. The actual mechanism of the low false-positive rates of the BEVL-BiFC system is still under investigation. We attempted to apply pBudCE4.1 in a Venus-based BiFC system. However, non-specific fluorescent protein fragment association was observed in the negative control, ΔFos/Jun and Grb2/K-Ras C185S. Thus, the mechanism of the low false-positive rate in the BEVL-BiFC system cannot simply be attributed to the pBudCE4.1 system independently expressing two genes with equivalent copy numbers in the same mammalian cells.

The post-translational modification (farnesylation, methylated and prenylcysteine) of K-Ras on its C-terminus is very important for its activity [23] and PM localization. Compared with the high-affinity interaction between the constitutively activated K-Ras 12v and RBD, we observed a weak interaction between inactive K-Ras and RBD in serum-starved cells. Non-farnesylated K-Ras (K-Ras C185S) has similar RBD interaction capability as K-Ras, indicating post-translational modification of K-Ras and its PM localization were not essential for stable association with the RBD. On the contrary, the interaction between Grb2 and K-Ras depended on the PM localization of K-Ras.

Due to low false-positives and dual protein expression, the BEVL-BiFC system is well suited to study PPI in the following applications. First, using a multi-color BiFC assay for the simultaneous investigation of three-pairs of PPIs, the BEVL-BiFC system with a bicistronic expression vector could potentially reduce the number of expression vectors required to express six fusion proteins from six plasmids to three. Second, the BEVL-BiFC might increase the reliability of the BiFC approach in screening unknown PPIs [24] owing to the low false-positive rate. Thirdly, the BEVL-BiFC system is also a potential tool to investigate PPIs in small animals owing to far-red emission of mLumin.

In summary, a novel BEVL-BiFC system was developed that decreased false-positives in BiFC assays. Using this BEVL-BiFC system, we provided direct evidence in individual cells that post-translational modification of K-Ras and its PM localization were not essential for the interaction between K-Ras and Raf-1, while the interaction of Grb2 and K-Ras depended on PM localization of K-Ras. The BEVL-BiFC system can be used for convenient and accurate investigation of PPIs.
