# Supplementary material for: Low False-Positives in an mLumin-Based Bimolecular Fluorescence Complementation System with a Bicistronic Expression Vector
Source: Sensors (Basel). 2014 Feb 19;14(2):3284–92. doi: 10.3390/s140203284 (PMC3958255; doi:10.3390/s140203284)
Supplement: Supplementary file 1 [file sensors-14-03284-s001.pdf]

OPEN ACCESS

sensors

ISSN 1424-8220
www.mdpi.com/journal/sensors

Supplementary Information

Low False-Positives in an mLumin-Based Bimolecular Fluorescence Complementation System with a Bicistronic. *Sensors*, 2014, *14*, 3284-3292

Shun Liu [1,†], Xiangyong Li [1,2,†], Jie Yang [1] and Zhihong Zhang [1,*]

[1] Britton Chance Center for Biomedical Photonics, Wuhan National Laboratory for Optoelectronics- Huazhong University of Science and Technology, Wuhan, Hubei 430074, China; E-Mails: liushun@ibp.hust.edu.cn (S.L.); xlxyongli@gmail.com (X.L.); yangjie@mail.hust.edu.cn (J.Y.)

[2] Department of Biology, Dezhou University, Dezhou, Shandong 253023, China

† These authors contributed equally to this work.

* Author to whom correspondence should be addressed; E-Mail: czyzzh@mail.hust.edu.cn); Tel.: +86-27-8779-2033; Fax: +86-27-8779-2034.

Supporting Information

Figure S1. Construct of the BEVL-BiFC vectors.

Figure S2. Confocal imaging the subcellular localization of CFP-RBD (a), EGFP-K-Ras (b) and EGFP-K-Ras C185S (c) in COS-7 cells.

Figure S3. Real-time monitoring the EGFR induced PM recruitment of Grb2 in living cells.

Supplemental Methods

Figure S1. Construct of the BEVL-BiFC vectors. (**a**) pBud-Ln-Fos(ΔFos)-Jun-Lc. Ln and Lc means N- and C- terminal of mLumin fluorescent protein. Ln-Fos or Ln-ΔFos fusion was inserted into *Hind III/BamH I*, bJun-Lc or Lc-bJun fusion was inserted by the sites *Not I/Mlu I*. (**b**) pBud-Ln-RBD-Lc-K-Ras. Ln-RBD or Ln-Grb2 fusion was inserted into *Hind III/BamH I* sites, Lc-K-Ras fusion was inserted by the sites *Not I/Mlu I*.

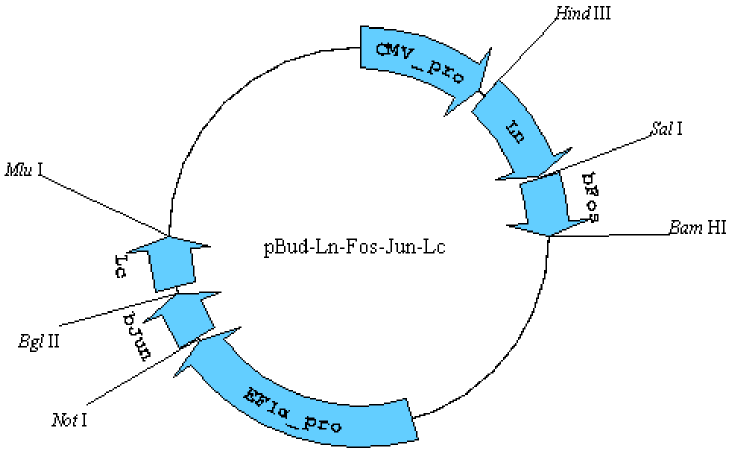

(**a**)

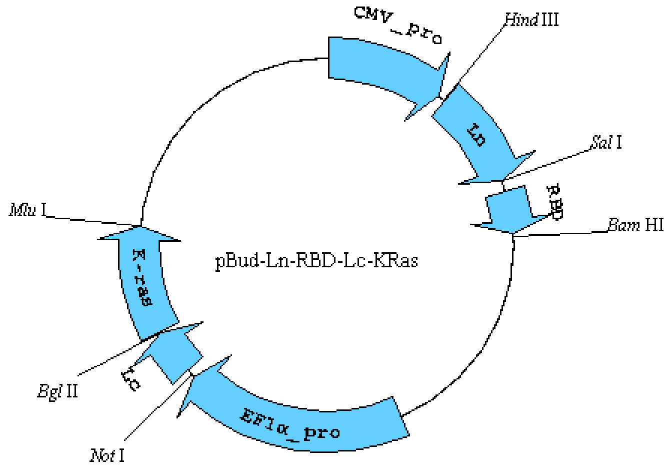

(**b**)

Figure S2. Confocal imaging the subcellular localization of CFP-RBD (**a**), EGFP-K-Ras (**b**) and EGFP-K-RasC185S (**c**) in COS-7 cells.

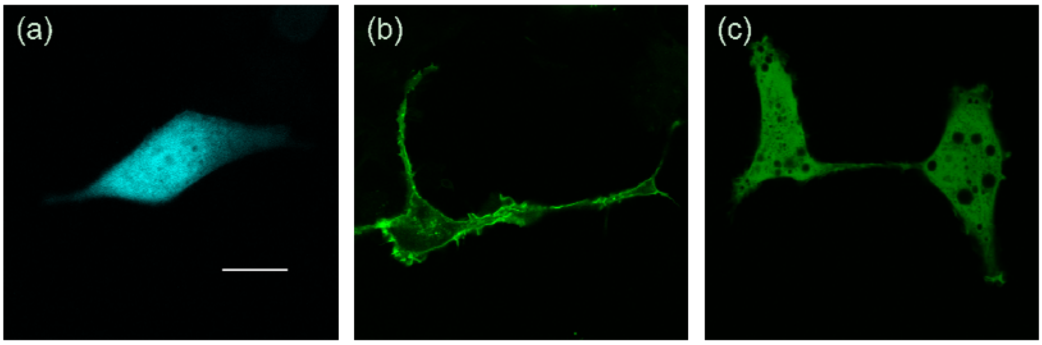

Figure S3. Real-time monitoring the EGFR induced PM recruitment of Grb2 in living cells. Grb2-EYFP located at the cytoplasm in inactivated cells and was recruited to PM in response to EGF stimulation. Co-localization of Grb2-EYFP and EGFR-mCerulean was visualized after EGF stimulation.

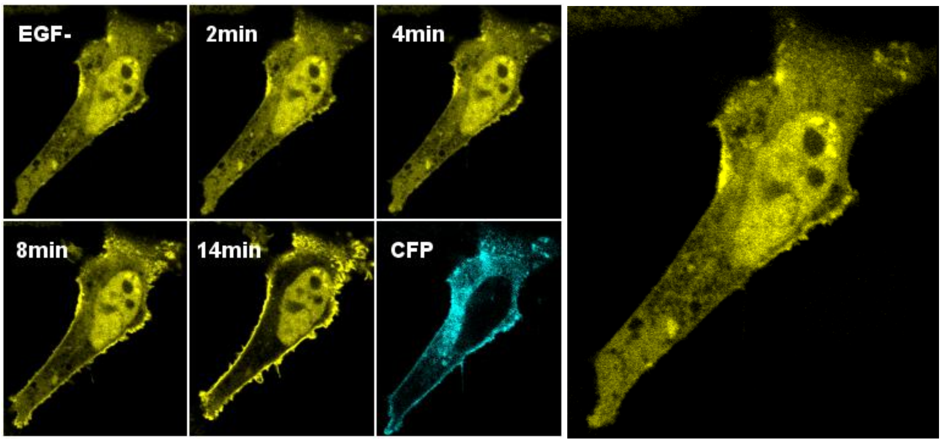

Supplemental Methods

S2.1. Construct of the Vectors

Plasmid EGFR-mCerulean was constructed by cloning EGFR into pmCerulean-C1; Grb2-EYFP was constructed by insert Grb2 into pEYFP-C1. All plasmid constructs were verified by sequencing. Plasmid concentrations were determined with a biophotometer (Eppendorf, Germany).

PCR primers used in this paper are list as follows:

primer	Sequence
RBD upstream	5'-ACTATCCGTGTTTTCTTGCCG-3'
RBD downstream	5'-CAGGAAATCTACTTGAAGTTC-3'
bFos upstream	5'-GGTCGTGCGCAGTCC-3'
bFos downstream	5'-ACCCAGGTCGTTCGGGA-3'.
bJun upstream	5'-ATGAAGGCGGAGAGGAAGCG-3'
bJun downstream	5'-AAACGTTTGCAACTGCTGC-3'
K-Ras upstream	5'-ATGACTGAATATAAACTTG-3'
K-Ras downstream	5'-CATAATTACACACTTTG-3'
Ln upstream	5'-ATGGTGTCTAAGGGCGAAG-3'
Ln downstream	5'-GTCAGCGGGGTACAGCATC-3'
Lc upstream	5'-GGCGGCCTGGAAGGCAGAG-3'
Lc downstream	5'-CTAATTAAGTTTGTGCCCCAG-3'
K-Ras G12V upstream	5'-ATGACTGAATATAAACTTGTGGTAGTTGGAGCTGTT-3'
K-Ras C185S downstream	5'-CATAATTACAGACTTTGTC-3'.

S2.2. Cells and Cells Transfection and Observation

For EGFR-mCerulean transfection, COS-7 cells were maintained in Dulbecco's modified Eagle's medium (DMEM) supplemented with 10% fetal bovine serum (FBS) at 37 °C in a humidified atmosphere containing 5% CO_2. 24 h before transfection, cells were seeded on 35-mm-diameter coverglass bottom dishes (MatTek Corporation, Ashland, MA) and grown to 80%–90% confluence. Cells were serum-starved for 4 hours until transfection. 100 ng each plasmids of EGFR- mCerulean and Grb2-YFP were transfected into cells used Lipofectamine™ 2000 (Invitrogen) according to the manufacturer's protocol. Cells cotransfected with EGFR-mCerulean and Grb2-YFP were cultured in DMEM without serum for 16 h after transfection. 100 ng/mL final concentration of EGF was added to cells before imaging.

S2.3. Fluorescent Microscopy and Image Processing

16 hrs after cotransfection, fluorescent signals of mLumin and Cerulean in COS-7 cells was detected using a wile-field fluorescence microscope with an RFP filter cube (Olympus, Japan, excitation 545-580HQ, dichroic DM600, emission 610IF) and CFP filter cube (excitation 425-445HQ, dichroic DM450, emission 410-510HQ), respectively. A Confocal Laser Scanning Microscopy (FV1000, Olympus, Japan) equipped with a HeNe-G laser and an UPlanSApo 100× oil-immersion objective lens (NA 1.40) was used to obtain high resolution images of PPIs. The BiFC mLumin fluorescence was excited at 543 nm and detected within a range from 580 nm to 680 nm. The GFP and YFP fluorescence were excited at 488 nm and 514 nm, respectively.
